# Rumen and Liver Fluke Infections in Sheep and Goats in Northern and Southern Germany

**DOI:** 10.3390/ani12070876

**Published:** 2022-03-30

**Authors:** Uta Alstedt, Katja Voigt, Miriam Carmen Jäger, Gabriela Knubben-Schweizer, Yury Zablotski, Christina Strube, Christoph Wenzel

**Affiliations:** 1 Clinic for Ruminants with Ambulatory and Herd Health Services, Centre for Clinical Veterinary Medicine, Ludwig-Maximilians-Universität München, 85764 Oberschleissheim, Germany; uta_alstedt@yahoo.de (U.A.); katja.voigt@lmu.de (K.V.); gknubben@med.vetmed.uni-muenchen.de (G.K.-S.); y.zablotski@med.vetmed.uni-muenchen.de (Y.Z.); 2 Labor ParaDocs, 85737 Ismaning, Germany; m.jaeger@laborparadocs.de; 3 Institute for Parasitology, Centre for Infection Medicine, University of Veterinary Medicine Hannover, 30559 Hannover, Germany; christina.strube@tiho-hannover.de

**Keywords:** paramphistomidosis, rumen flukes, *Calicophoron daubneyi*, fasciolosis, *Fasciola hepatica*, dicrocoeliosis, *Dicrocoelium dendriticum*, trematodes, small ruminants, risk factors

## Abstract

**Simple Summary:**

Little is known about the distribution of rumen and liver flukes in sheep and goats in Germany or about the occurring rumen fluke species. These fluke infections can be detected by the parasite’s eggs in the host animal’s feces. Therefore, fecal samples from 223 sheep farms and 143 goat farms from northern and southern Germany were examined. The eggs of rumen flukes were detected on 2.2% of the samples, the eggs of common liver flukes on 2.7%, and the eggs of small liver flukes on 21.1% of the examined sheep farms. The rumen flukes were identified as the emerging species *Calicophoron daubneyi*. No rumen fluke eggs were detected on any of the goat farm samples, but common and small liver fluke eggs were detected in 5.6% and 7.0% of the goat herds, respectively. Differences in the geographical distribution of rumen and liver flukes between and within the two regions were identified. Rumen flukes were more frequently found in the north, while the two liver fluke species were more frequently found in the south of Germany. Sheep sharing the pasture with other ruminants were more likely to be infected with rumen flukes.

**Abstract:**

Paramphistomidosis has recently been identified as an emerging parasitosis in Europe. This study estimated the prevalence of rumen flukes, *Fasciola hepatica* and *Dicrocoelium dendriticum*, in small ruminants in Germany and identified occurring rumen fluke species and potential predictors for fluke infections. Pooled fecal samples from 223 sheep farms and 143 goat farms in northern and southern Germany were examined by the sedimentation technique, and molecular species identification was performed on rumen-fluke-positive samples. In sheep, a flock prevalence of 2.2% was detected for rumen flukes. *Calicophoron daubneyi* was identified on four of five positive farms, while species identification failed in one flock. No rumen fluke eggs were detected in the examined goat herds. *F. hepatica* eggs were detected in 2.7% of the sheep flocks, while the herd prevalence was 5.6% in goats. Higher prevalence values of 21.1% (sheep) and 7.0% (goats) were observed for *D. dendriticum*. Mixed grazing with other ruminants and previously identified infections with rumen flukes and/or *F. hepatica* were identified as predictors for paramphistomidosis. The distribution of the three trematode species followed a geographical pattern associated with conditions favoring the relevant intermediate hosts. *C. daubneyi* is an established parasite in German sheep at a currently low prevalence.

## 1. Introduction

While infections of *Fasciola hepatica* (common liver fluke) and *Dicrocoelium dendriticum* (small liver fluke, lancet fluke) are well-established in European small ruminant flocks [1], paramphistomes (rumen flukes) and particularly *Calicophoron daubneyi* are increasingly being diagnosed in a variety of European countries. High rumen fluke prevalence was originally mostly reported in (sub)tropical regions [2,3,4]. Paramphistomidosis has thus been considered an emerging parasitic disease in Europe [5], and species identification revealed *C. daubneyi* as the dominant rumen fluke species in European cattle, sheep, and goats in the last fifteen years. In contrast, the other rumen fluke species recently reported in Europe, *Paramphistomum leydeni*, has only rarely been detected in cattle, sheep, and wild ruminants [5,6,7,8,9,10].

Rumen flukes have a diheteroxenous life cycle similar to *F. hepatica*. Both need air-breathing freshwater snails for their development [11]. *F. hepatica* and *C. daubneyi* share the same intermediate host, the amphibious snail *Galba truncatula* [12]. Hence, co-infections in the intermediate and final hosts are possible [13,14]. Favorable primary habitats of *G. truncatula* are drainage ditches, ponds, and the watersides of slow-moving streams [15,16]. The final hosts of rumen flukes are domestic and wild ruminants [17], as well as new-world camelids [18]. Infection follows the ingestion of encysted metacercariae on plants or floating metacercariae in water [19]. Rumen fluke metacercariae excyst in the small intestine, followed by retrograde migration towards the rumen [20,21], where they mature into adults and start egg production. Intestinal paramphistomidosis may cause clinical signs such as diarrhea, weight loss, submandibular oedema, and even death of the animals [22,23,24], while the ruminal phase is usually clinically inapparent. Atrophy of the ruminal papillae can, however, be seen in postmortem examinations [25].

*F. hepatica* is a well-known trematode, which causes severe clinical disease and production loss in the livestock industry across Europe [26,27]. Following the ingestion of encysted metacercariae, the life cycle includes an abdominal and hepatic migration of juvenile flukes before the adults settle in the bile ducts [28]. The disease can be (sub)acute, involving reduced feed intake, lethargy, anemia, and sudden death [27]. Chronic fasciolosis is often associated with weight loss, reduced wool quality and milk production, anemia, submandibular oedema, and ascites [27,29]. Both forms can also be associated with secondary reproductive failure [30,31].

*D. dendriticum* has a triheteroxenous life cycle including terrestrial snails as the first and ant species as the second intermediate host. Dicrocoeliosis is mostly prevalent in areas with dry pastures that favor the development of the intermediate hosts [32]. However, these parasites are also found in regions with high precipitation, due to both the large variety of terrestrial snail species serving as first intermediate hosts, and the ubiquitous occurrence of ants [33]. The final hosts are infected by ingestion of metacercariae-carrying ants. Excysted metacercariae migrate from the small intestine via the ductus choledochus into the hepatic bile ducts and the gall bladder [28]. The relevance of dicrocoeliosis is often underestimated due to its often subclinical course in domestic ruminants, and because clinical signs can be masked by other parasitic infections [1,34]. Infection, therefore, often remains untreated. However, the importance of dicrocoeliosis for sheep health has recently been highlighted [35], and it is assumed that the infection results in production losses [36].

Final hosts of both liver fluke species are ruminants, new world camelids, horses, pigs, and other mammals [28]. Both have zoonotic potential following the ingestion of metacercariae [37].

Increasing rumen fluke prevalence has been observed for cattle and sheep in Europe in recent years. Reported flock prevalence values for sheep were highly variable between different studies and regions and ranged from 7.9% in southern Italy to 77.3% in Ireland [8,38]. Only three studies so far have detected rumen fluke infections in European goats. Published reports of rumen fluke infections in German sheep or goats are rare. Table 1 provides a detailed overview on rumen fluke prevalence or observed infections in sheep and goats in various European countries on a flock or individual animal level. Common liver fluke and lancet fluke infections have been long established in Europe, but prevalence studies from Germany in particular are rare, with previous studies limited to low flock or animal numbers, certain regions, or specific farm types. To provide a European context, Table 2 and Table 3 show literature reviews of reported prevalence values or observed infections for *F. hepatica* and *D. dendriticum*, respectively, in both small ruminant species across Europe.

Potential risk factors for patent rumen fluke infections were studied across various regions and countries. A Welsh study including sheep and cattle identified more sunshine hours, regular treatment against *F. hepatica*, higher Ollerenshaw-indexes (a score to predict the appearance of *F. hepatica* based on the amount of precipitation, evaporation, and rainy days per month), larger herd sizes, the presence of streams, boggy habitats, and higher precipitation during the summer as positive predictors for infections with *C. daubneyi*. Lower winter temperatures and lower rainfall during the summer were identified as negative predictors [39,52]. In Ireland, sheep grazing on lowland pastures were more likely to be infected with rumen flukes than sheep grazing on mountain pastures. Furthermore, sheep sharing the pasture with other livestock, mostly cattle, had a higher risk of infection [8]. Other Irish studies showed that higher prevalence was detected during the rainy winter months [53,54]. The presence of streams and springs on the pasture was also identified as a risk factor in southern Italy [40]. In a different Italian study, an altitude of 500 to 1500 m was identified as a positive predictor, in addition to the presence of streams and brooks [38].

There is currently only limited data available on the prevalence of trematode infections in small ruminants in Germany. The detection of rumen fluke infections, in particular, has so far been limited to anecdotal evidence and two publications identifying individual positive farms in the federal state of Schleswig-Holstein [41,44]. The more widespread occurrence and distribution of *F. hepatica*, which shares its intermediate host with *C. daubneyi*, as well as recent findings of rumen fluke infections in German cattle [6], led to the assumption that paramphistomidosis may also be spreading in small ruminant flocks in Germany. The present study was therefore conducted to estimate a flock/herd prevalence of paramphistomidosis in participating northern and southern German sheep and goat farms from the federal states of Lower Saxony and Bavaria using fecal examination to identify the rumen fluke species involved and to concurrently record the prevalence of *F. hepatica* and *D. dendriticum*. In addition, it aimed to determine the potential risk factors associated with the occurrence of the three trematodes on the examined farms.

## 2. Materials and Methods

### 2.1. Sample Population and Sample Collection

This study was undertaken between July 2019 and April 2021. The federal states of Bavaria and Lower Saxony were chosen because they are the two largest federal states and together amount to a third of the total area of Germany, with large sheep and goat populations in both regions. More than a third of the sheep and goat farms in Germany are situated in these two federal states [55]. Bavaria, followed by Lower Saxony, has the highest percentage of farmland of all 16 German federal states [56]. Both states vary geographically and climatically. Bavaria is located in southern Germany, and, while its northern part is relatively dry, the southern part includes some of the regions with the highest precipitation levels recorded in Germany and reaches into mountainous, Alpine areas. Lower Saxony is located in northern Germany between the North Sea to the northwest and a low mountain range at its southeastern border.

The planned sample size (number of farms) for a robust statistical analysis was calculated [57,58] based on the number of sheep and goat farms in the two regions and an assumption of a low rumen fluke prevalence of approximately 5% in both species. This was based on recent studies in German cattle [6,59,60] and little or no findings in sheep and goats in previous studies [41,48,50]. To achieve a 95% confidence level and a precision of 5% for a sheep population of 5140 Bavarian flocks and 2167 Lower Saxonian flocks [55], the calculated sample size was 72 and 71 sheep flocks, respectively. For a goat population of 3041 herds in Bavaria and 770 herds in Lower Saxony, the calculated sample size was 72 and 67 goat herds, respectively.

Diagnostic sample submissions for routine health monitoring were used. The farm selection was, therefore, based on voluntary participation. In return for consent being given for their samples and information being used in the study, additional coproscopical examination for gastrointestinal nematodes was offered to the participants free of charge (results not shown). The samples were submitted by veterinarians, local animal health services, or the farmers themselves following advertisements via veterinary organizations, regional and national breeding associations, and professional journals. Inclusion criteria for the participation of farms were (a) location in Bavaria or Lower Saxony, (b) access to pasture (at least temporarily), and (c) age of the sampled animals >3 months. For participating farms, the minimum flock size was set at 15 animals. Difficulties in recruiting sufficient goat herds, particularly in Lower Saxony, led to the inclusion of smaller herds with less than 15 eligible animals. The minimum herd size was thus set at three eligible animals for goats.

Interested farmers or their veterinarians were sent a sampling kit, which contained three sample containers, a pair of gloves, an instruction sheet, a questionnaire, and a pre-paid, pre-addressed return label for sample delivery to the Clinic for Ruminants, Ludwig-Maximilians-Universität München, Germany. The sheep farmers were asked to submit three pooled samples, each from five different sheep. Sampling criteria were identical for larger goat herds. In herds with less than 15 eligible goats each animal was sampled and a minimum of three individual fecal samples was set per sample pool.

### 2.2. Coproscopical Examination for Fluke Eggs

To detect patent fluke infections each pooled sample was examined individually by a sedimentation technique according to Deplazes et al. [61]. Feces from each container were homogenized prior to examination using a combined spatula–spoon. An average amount of 9.9 g feces was used, ranging from 1 to 10 g depending on the amount of material submitted. This amount was further homogenized using a spoon, mixed with water, and washed through a 1500 µm mesh sieve into a 500 mL glass beaker using cold tap water. The supernatant was decanted after 15 min of sedimentation. This process was repeated until the supernatant was clear. The sediment was carefully resuspended and poured through a 300 µm mesh sieve into a petri dish. The whole sediment was then transferred successively into a counting dish, and all trematode eggs were counted using a stereomicroscope (Labophot-2, Nikon, Japan). A flock was defined as positive if at least one egg of the respective trematode species was detected in any of the examined samples. Liver and rumen fluke eggs were differentiated by color and size [34,62]. The eggs were counted and the egg counts were classified semi-quantitatively as “low” (1–10 eggs), “medium” (11–30 eggs), and “high” (>30 eggs). For rumen and common liver fluke eggs, the results were also assessed quantitatively by dividing the number of fluke eggs counted by the fecal weight to calculate the eggs per gram feces (epg). The numbers of lancet fluke eggs were counted until 31 eggs per sediment was reached. Each sample containing more than 30 *D. dendriticum* eggs was classified semi-quantitatively as “high” without further counting.

### 2.3. Molecular Species Identification

Rumen fluke eggs isolated from the sediment were incubated with 90 µL DirectPCR^®^ Lysis Reagent Cell (Peqlab, Erlangen, Germany) and 10 µL Proteinase K (Peqlab, Germany) for 16 h at 55 °C, followed by 45 min at 85 °C to isolate genomic DNA. The subsequent PCR amplified the ITS-2 region and flanking 5.8S and 28S rDNA sequences. The primers ITS-2For (5′ TGTGTCGATGAAGAGCGCAG 3′) and ITS-2Rev (5′ TGGTTAGTTTCTTTTCCTCCGC 3′) were used [8,63]. The reaction was carried out in a 50 µL reaction containing 0.5 µL HOT FIREPol^®^ DNA Polymerase (5 U/µL) (Solis BioDyne, Tartu, Estonia), 5 µL buffer B (10×), 1 µL dNTP (10 mM each), 3 µL MgCl_2_ (25 mM), 1 µL of each primer, 28.5 µL double-distilled water, and 10 µL of the extracted DNA. The thermocycling conditions were an initial denaturation at 95 °C for 15 min, followed by 40 cycles at 95 °C for 30 s, 53 °C for 1 min, 72 °C for 45 s, and a final elongation at 72 °C for 10 min. The PCR products were separated on a 1% agarose gel and subsequently Sanger-sequenced (Seqlab Sequence Laboratories Göttingen, Germany). Obtained nucleotide sequences were compared to published sequences in the NCBI GenBank database [59]. No molecular species identification was performed for liver fluke eggs, as *F. hepatica* and *D. dendriticum* are the only occurring species in Europe, and egg morphology is thus sufficient for reliable identification [1,28].

### 2.4. Questionnaire

Data on farm management and farm structure were collected by questionnaire. The multiple-choice survey was based on 11 semi-closed questions about (a) farm location as identified by the post code, (b) farm management: flock size, flock purpose, and grazing management, (c) detailed information on pasture including potential intermediate host habitats and shared pastures with other livestock, and (d) previous coproscopic results, last anthelmintic treatment, and type of anthelmintic product used.

### 2.5. Statistical Analyses

The replies to the questionnaire were coded, entered, and sorted using Microsoft Excel 2019 (Microsoft Corporation, Redmond, WA, USA). The statistical analyses were performed in R version 4.0.3. (The R Foundation for Statistical Computing, Vienna, Austria) [64]. The 95% confidence intervals (CI) for the prevalence values of patent rumen and liver fluke infections were calculated using the Wald-test. The Chi Square test was used to study the association between the small ruminant species and the use of fasciolicides, and between the two federal states and the use of fasciolicides. Simple logistic regressions were performed to study the influence of different predictors (species; federal state; potential habitat for *G. truncatula* (for rumen and common liver fluke); dry pastures (for *D. dendriticum*); other potential final hosts on the farm; and previous coproscopic results) on the occurrence of patent rumen or liver fluke infections on the farms. For statistical analyses, other livestock was categorized as “ruminants”, “equids”, and “camelids”. Camelids were subsequently excluded from the statistical analysis due to low case numbers. Pastures containing a ditch, stream, or pond or described as (temporarily) wet were assumed to contain a possible habitat for *G. truncatula*. Prior infections with rumen and/or common liver flukes were summarized as “prior infections with *G. truncatula*-dependent trematodes”. *p*-values ≤ 0.05 were considered statistically significant.

## 3. Results

### 3.1. Descriptive Analysis

A total of 223 sheep flocks (*n* = 666 pooled samples) and 143 goat herds (*n* = 392 pooled samples) were included in the study. Farms that provided less than the requested number of pooled samples were also included in the final analysis (*n* = 3 sheep and *n* = 3 goat farms). Three sheep farmers only collected two pooled samples of a total of 10 sheep instead of the requested three pools from 15 animals. Two goat keepers provided only one pooled sample, and another provided only two instead of three. Therefore, a total of 220 sheep (98.7%) and 140 goat farmers (97.9%) complied with the sampling instructions. Participation exceeded the planned sample size in Bavaria, with 144 participating sheep farms and 80 goat farms. From Lower Saxony, the planned sample size was met for sheep flocks (*n* = 79) but narrowly missed by four farms for goats (*n* = 63). Of the 143 goat farmers, 37 (25.9%) kept less than fifteen eligible goats.

The participating farms showed a great variety regarding farm type, flock size, and breed. The flock or herd size was categorized based on the number of breeding ewes or adult female goats as “3–15”, “16–50”, “51–200”, “201–500”, “501–800” and “>801”. The majority of participating sheep flocks (36.8%) kept between 16 and 50 ewes, and the flock size ranged from 5 to 1700 ewes. For goats, the predominant herd size was 3–15 does (44.5%), with a minimum of three and a maximum of 280. Forty-seven different sheep breeds were kept on the examined farms. Merino and Merino crosses (23.9%) were the predominant breed, followed by German Blackhead Mutton and their crosses (10.9%). Within the 18 different goat breeds, Alpine Goats and their crosses (25.8%) were the most frequent breeds, followed by Boer Goats and their crosses (18.5%) and Saanen Goats (17.4%).

Over 80% of both species were kept for agricultural purposes, with the other purposes including pedigree breeding, hobby flocks, and zoos (Table 4). Sheep were most commonly grazed in a rotational pasture system (52.6%), whilst the majority of goats were grazed on permanent pastures (38.0%). Grazing systems for sheep also included permanent (12.5%) and strip-grazed pastures (14.9%), migratory flocks (10.4%), and stationary shepherding systems (9.7%). Goats were also kept on rotational pastures (37.0%), strip-grazed pastures (18.8%), in migratory herds (2.6%), stationary shepherding systems (1.6%), and on community pastures (2.1%).

Regarding pasture conditions, 52.5% of sheep and 44.1% of goat farmers indicated having (temporarily) wet pastures. The remaining farms were assumed to be dry. In addition, 9.4% of sheep and 4.9% of goat pastures which were not declared as (temporarily) wet contained a ditch, pond, or stream. A potential habitat for *G. truncatula* was therefore present on 61.9% of all sheep farms (56.3% of Bavarian and 27.2% of Lower Saxonian farms) and on 49.0% of the goat farms (45.0% of Bavarian and 54.0% of Lower Saxonian goat farms) according to the questionnaire results. Of all the farms with a potential habitat for *G. truncatula* (sheep: *n* = 138; goats: *n* = 70), 58.7% (sheep) and 51.4% (goats) were located in Bavaria.

Pastures were shared with one or more other livestock species (ruminants, equids, camelids) on 17.9% of sheep farms and 38.5% of goat farms. Sheep pastures were co-grazed by goats (40.9%), cattle (34.1%), horses (20.5%), llamas (2.3%), and donkeys (2.3%), while other livestock species on goat pastures included horses (36.1%), sheep (32.8%), cattle (27.9%), llamas (1.6%), and donkeys (1.6%).

Rumen flukes or common liver flukes had not previously been diagnosed on the majority of the participating sheep farms (69.1%) and goat farms (79.0%). Fourteen sheep farmers and one goat farmer reported previously identified rumen fluke infections. Of these, both rumen and common liver fluke infections had been diagnosed in 12 sheep flocks. Past infections with *F. hepatica* (including the farms with both diagnoses) were known in 40 sheep and 13 goat farms. No information was available on previously diagnosed *D. dendriticum* infections.

Anthelmintic treatment(s) up to six months prior to sample submission had been carried out on 61.9% (138/223) of sheep and 51.7% (74/143) of goat farms. Five sheep and six goat farmers did not provide any information on anthelmintic treatments. The remaining farmers had not carried out any treatments during this time period, and their flocks were classified as “not dewormed”. This included 36.7% (82/223) of sheep farms and 44.1% (63/143) of goat farms. The difference in treatments between the two species was statistically significant (*p* = 0.007). Thirty-nine sheep farms (17.6%) had used fasciolicides (12 albendazole, 2 closantel, 16 closantel + mebendazole, 8 triclabendazole + moxidectin, and 1 triclabendazole). Fasciolicides had also been applied on 11 goat farms (7.7%) (6 albendazole, 1 closantel + mebendazole, 3 triclabendazole + moxidectin, and 1 triclabendazole). None of the farmers had carried out any specific treatments against rumen flukes in the past six months. Further anthelmintic treatments were targeted at gastrointestinal nematodes or cestodes on 97 sheep farms and 63 goat farms. The use of fasciolicides including albendazole was slightly more frequent in Lower Saxony than in Bavaria (16.3% vs. 12.1%). This difference was, however, not statistically significant. An opposite trend was observed for the use of albendazole alone, the only anthelmintic drug effective (in increased dosage) against *D. dendriticum* (Lower Saxony: 3.5%, Bavaria: 5.8%). Table 5 summarizes the recent anthelmintic treatments on farms with and without diagnosed trematode infections.

### 3.2. Rumen and Liver Fluke Prevalence Values

**Figure 1:**
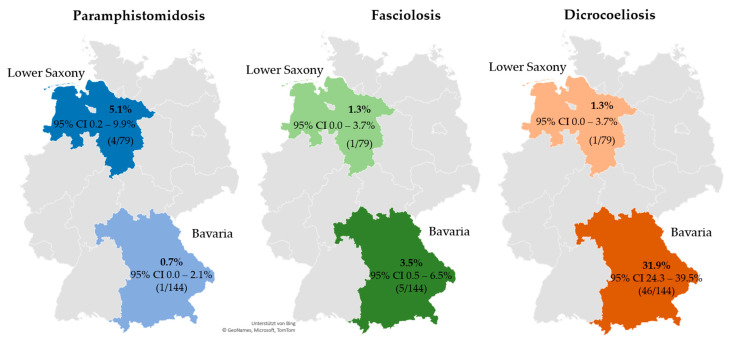
Prevalence of patent paramphistomidosis, fasciolosis and dicrocoeliosis, including 95% CI for sheep farms in Bavaria and Lower Saxony (co-infections included). Abbreviations: CI, confidence interval.

**Figure 2:**
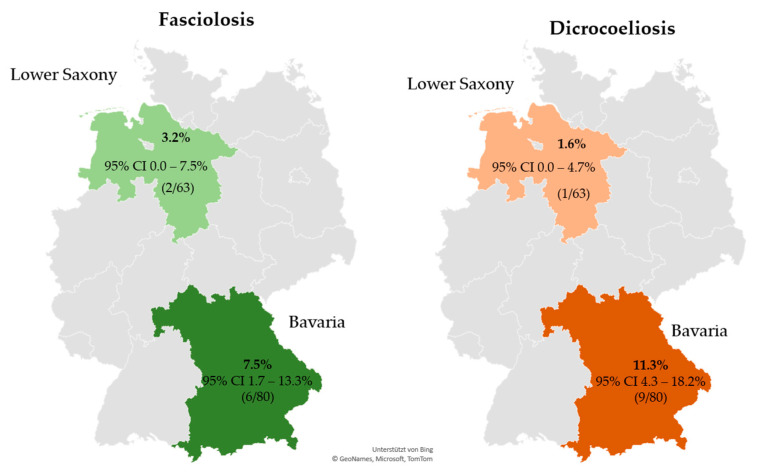
Prevalence of patent fasciolosis and dicrocoeliosis, including 95% CI for goat farms in Bavaria and Lower Saxony. Note that no patent paramphistomidosis was diagnosed in the examined goat herds. Abbreviations: CI, confidence interval.

**Figure 3:**
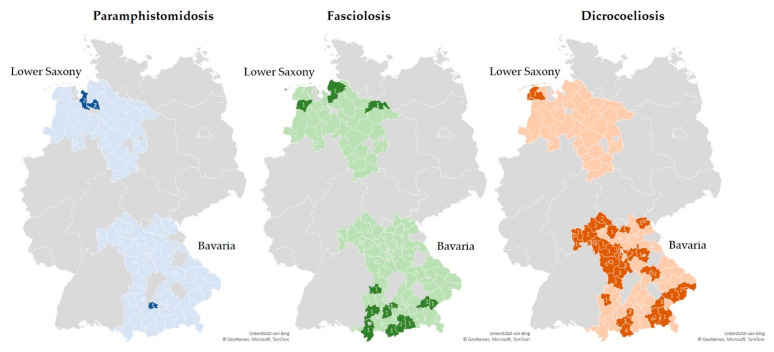
Distribution and numbers of rumen fluke, *F. hepatica*, and *D. dendriticum*-positive sheep farms and goat farms by administrative district within the federal states of Bavaria and Lower Saxony. Light color: district with sample submission(s) but no egg detection; dark color and number: district with detection of respective trematode eggs and number of positive small ruminant farms from district.

The estimated prevalence of patent rumen fluke infections in sheep flocks was 2.2% in the two investigated German federal states (95% CI 0.3–4.2%, 5/223). A higher prevalence was observed in Lower Saxony (5.1%) compared with Bavaria (0.7%) (odds ratio (OR): 5.07; *p* = 0.076). Details are presented in Figure 1 and Table 6. No rumen fluke eggs were detected in goat herds in either of the two federal states. The prevalence of patent *F. hepatica* infections on the examined sheep farms and goat farms was estimated at 2.7% (95% CI 0.6–4.8%, 6/223) and 5.6% (95% CI 1.8–9.4%, 8/143), respectively, with no significant difference between the two regions for either species. Details are provided in Figure 1 and Figure 2. Co-infection with rumen and common liver flukes was diagnosed on one sheep farm in Bavaria (0.45%, 95% CI 0.0–1.3%). For *D. dendriticum*, the prevalence of patent infections was estimated at 21.1% (95% CI 15.7–26.4%, 47/223) for sheep flocks and 7.0% (95% CI 2.8–11.2%, 10/143) for goat farms. Further details are provided in Figure 2. Sheep farms and goat farms in Lower Saxony had significantly lower odds of being infected with *D. dendriticum* than farms located in Bavaria. Detailed results are listed in Table 6. Figure 3 shows the geographical distribution of patent infections with the three trematodes within the two federal states by administrative district.

Eight samples from five sheep farms were found to be positive for rumen fluke eggs. Seven of these contained up to 10 eggs (semi-quantitatively classified as low), while one sample was classified as medium quantity (11 eggs). The average rumen fluke egg count was 0.3 epg (range, 0.1–1.1 epg) in the positive samples. *F. hepatica* was identified in 15 samples from 14 farms. Again, the majority (14/15) of these contained only low egg numbers (sheep: 5 samples; goats: 9 samples), and only one sheep sample was categorized as medium quantity (22 eggs). The average egg count in positive *F. hepatica* samples was 0.5 epg (range, 0.1–2.2 epg) in ovine samples and 0.4 epg (range, 0.1–0.9 epg) in caprine samples. Of all samples positive for *D. dendriticum* eggs, 76.2% of ovine (*n* = 74/97) and all caprine samples contained low to medium quantities, and the eggs were counted completely. *D. dendriticum* egg counts were low in 50 sheep samples and 14 goat samples, while 28 ovine samples and two caprine samples contained up to 30 eggs (medium quantity). The average egg count in the goat samples was 0.5 epg (range, 0.1–1.3). The remaining 23.7% of sheep samples (*n* = 23/97) contained more than 30 eggs and were semi-quantitatively classified as “high”.

### 3.3. Species Identification of Rumen Fluke Eggs

Molecular species identification was attempted for each rumen-fluke-positive sample (*n* = 8 originating from 5 farms). Four samples from four farms (Bavaria: *n* = 1, Lower Saxony: *n* = 3) resulted in PCR products of the expected size of approximately 440 bp and were identified as *C. daubneyi* by sequencing. The nucleotide sequences obtained showed 99.4–100% identity (query cover: 100% each) of the published top-hit *C. daubneyi* sequence (GenBank accession number KP201674). The remaining samples (*n* = 4 samples, including one from an additional farm) could not be identified because of insufficient DNA material due to very low egg quantities. The rumen fluke species present on one farm in Lower Saxony therefore remains unidentified.

### 3.4. Statistical Analyses

**Figure 4:**
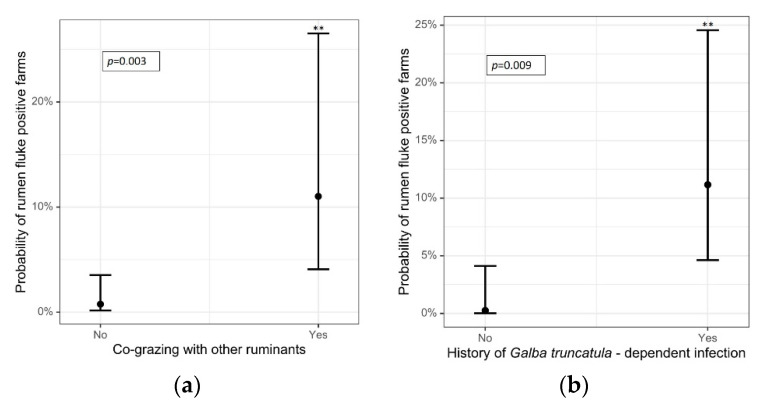
Predicted probabilities of patent rumen fluke infections in sheep in relation to (**a**) co-grazing with other ruminants (*p* = 0.003) and (**b**) history of prior *G. truncatula*-dependent trematode infections on the farm (*p* = 0.009). The error bars indicate the 95% confidence intervals. **: *p*-value < 0.01.

Potential predictors for patent rumen and liver fluke infections were evaluated by simple logistic regressions. The pasture of each rumen-fluke-positive flock contained a ditch, pond, or a stream and was described as (temporarily) wet according to the questionnaire results, thus providing a potential habitat for *G. truncatula*. However, this was not statistically significant (OR: 8.41; *p* = 0.138). A positive relationship was determined between rumen fluke infection and pastures shared with other ruminants (*n* = 4 of 5 positive flocks; OR: 16.19, *p* = 0.003; Figure 4a). These included only cattle on two farms, cattle plus horses on another, and goats on the fourth. These goats were also examined and were negative for rumen and liver flukes. Each rumen fluke-positive farm stated a previously diagnosed rumen or common liver fluke infection in the questionnaire. One farmer reported a prior *F. hepatica* infection, another a prior rumen fluke infection, and three farmers reported previous infections with both rumen and common liver flukes. Sheep flocks with a history of rumen fluke and/or *F. hepatica* infections had higher odds of being positive for rumen fluke eggs than sheep farms with no history of these trematodes in their flocks (Figure 4b). Further details of the simple logistic regressions are presented in Table 7.

A potential habitat for *G. truncatula* (sheep: 5/6; goats: 6/8) was present on most of the 14 farms positive for *F. hepatica* according to the questionnaire results. However, this result showed no statistical significance (sheep: OR: 2.42, *p* = 0.327, goats: OR: 2.71, *p* = 0.180). Similarly, co-grazing with other livestock was not associated with increased odds of testing positive for *F. hepatica* in our sample set. This applied to both species. While a history of *F. hepatica* infections, as well as a history of infections with *G. truncatula*-dependent trematodes, was associated with higher odds of patent fasciolosis (*p* = 0.011 and *p* = 0.014, respectively) in goat herds, no such relationship was observed in sheep flocks. Details of these analyses are presented in Table 8.

Based on the questionnaire results, dry pastures were assumed on 49.0% and 60.0% of *D. dendriticum*-positive sheep farms and goat farms, respectively. No statistical relationship was seen between dicrocoeliosis in sheep flocks and goat herds and environmental factors such as the pasture conditions (*p* = 0.803, *p* = 0.511) or shared pastures with other livestock. Details of the statistical analyses are presented in Table 9.

## 4. Discussion

The rumen fluke prevalence on sheep farms and goat farms in northern and southern Germany was lower than expected and considerably lower than in recently published studies from other European countries [8,39]. It was also below the prevalence of 5.5% recently identified in German cattle by our research group using identical methods (Lower Saxony: 10.9%, Bavaria: 4.4%) [6]. In comparison to cattle, a lower rumen fluke prevalence in small ruminants has also been observed in most other European studies which simultaneously studied or conducted comparable studies in all these species [10,38,39,42,44,54,65]. The detected prevalence of patent *F. hepatica* infections was similar to the percentages of positive farms or samples identified in previous German studies [41,48,50], despite considerable differences in farm numbers, representation, and farm or sample selection. Similar to rumen flukes, a comparable study concerning cattle recently revealed a higher prevalence in bovine samples; the nationwide *F. hepatica* prevalence was estimated to be 9.5% on German cattle farms (Lower Saxony: 6.5%, Bavaria: 16.1%) [6].

Statistical analyses in the present study were hampered by the low numbers of rumen and common liver fluke positive farms. In addition, the assessment of pasture conditions by questionnaire reflects the farmers’ perceptions and may thus be subjective and possibly inaccurate. Both these limitations can serve as an explanation for the unexpected lack of statistically significant associations between pasture conditions and detected patent infections with the three trematode species. A farm history of rumen fluke and/or *F. hepatica* infection was associated with a higher risk of rumen and common liver fluke infections in the present study, an observation also made on Welsh farms [52]. This fact must be considered as a potential bias of the study.

The observed relationship between rumen-fluke-positive farms and shared pastures with other ruminants is in accordance with the results of other authors, who suggested that infected cattle grazing the same pasture spread the parasite eggs and therefore act as a predictor [8,65]. The similar geographical distribution of rumen fluke infections in German cattle also supports this assumption [6].

Despite recruiting representative farm numbers for the sheep and goat populations in the examined federal states (except for goat herds in Lower Saxony), a truly representative farm selection was not possible, as the study relied on voluntary participation and the submission of diagnostic samples. An attempt was made to avoid a bias related to farm participation by offering an additional, free of charge fecal examination for gastrointestinal nematodes, a universal problem in sheep and goat farming and thus relevant to nearly any animal keeper. However, a potential bias cannot be ruled out entirely. Farmers with a history of trematode problems may have been more likely to take part in a trematode study, thus leading to an overestimation of prevalence, or vice versa, i.e., farmers with knowledge of trematode infections on their farms may have been less inclined to submit additional samples, thus potentially leading to an underestimation of prevalence. Sample submissions, however, covered a wide range of farm types and sizes, correctly reflecting the diversity and predominantly small-scale structure of German sheep and goat husbandry [55]. To date, this is the most comprehensive study that has been undertaken to assess trematode prevalence in Germany, although only samples from Lower Saxony and Bavaria were included. The trematode prevalence in other federal states therefore remains unknown. The two selected federal states can, however, be considered representative for the whole country in terms of geographic and climatic variation [66], as well as in terms of livestock population [55,56,67].

A comparison of our results to previous European studies is difficult due to the great variation in the conditions for participation, sampling, examination methods, and climate and the lack of representativeness of the selected areas or farm types [10,38,39,40,42,43,46,48,50]. For example, a rumen fluke prevalence of 8.0% was reported in the Netherlands, but the authors discuss that this may be an overestimation due to a bias in participating flocks located in areas with a high risk of *F. hepatica* infections [10].

A history of rumen fluke infections in their flocks was mentioned by 6.3% of sheep (14/223; Bavaria: *n* = 3, Lower Saxony: *n* = 11) and 0.7% of goat farmers (1/143; in Bavaria) in the questionnaire. This exceeds the percentage of positive farms identified by coproscopical examination and may be an indication that the true prevalence of rumen fluke infections may be higher than detected, or that these farms have since established effective control measures. Detailed information on the date and diagnostic methods leading to previous diagnoses was not recorded in the questionnaire, but some farmers mentioned post-mortem diagnoses following slaughter. It is possible that the applied sedimentation technique was not sufficiently sensitive to detect low egg counts and thus contributed to a potential underestimation of the prevalence in this coproscopical study. Sedimentation techniques are, however, a common method for the detection of rumen and liver fluke eggs [1,61] and have been widely used in comparable studies [8,9,14,39,42,44,46,54]. Further potential reasons for the difference between historically reported and coproscopically identified rumen fluke infections are examinations during the prepatent period [68], or very low egg excretion, possibly due to (still) low adult fluke burdens. Ploeger et al. [10] reported that rumen fluke burdens of up to 500 adult flukes can result in negative fecal egg counts. Anthelmintic treatment prior to sample collection is another potential reason leading to a possible underestimation of prevalence by coproscopical methods. The only effective anthelmintic compound available for the treatment of rumen flukes in Germany is oxyclozanide. However, its effectiveness on immature flukes has not been completely clarified [69,70]. According to the questionnaire data, no treatments with this drug had been carried out on the participating farms within six months prior to sample submission. The partial elimination of rumen flukes in cattle has, however, also been described following treatment with closantel [71], but its effectiveness on fecal egg reduction was only 0–81% in sheep [69]. Closantel was used on 18 sheep farms and one goat farm in the six months prior to sample submission. Therefore, partial fluke elimination could have resulted in low egg excretion and negative coproscopical examinations despite the presence of the parasite on the farm.

A major difficulty of this study was the recruitment of adequate numbers of goat farms with sufficient eligible animals contributing to the pooled fecal samples, especially from Lower Saxony. Goat farming is particularly small-scale in Germany, with 86.7% of all German goat farms (88.6% of Bavarian herds, 89.7% of Lower Saxonian herds) keeping between one and 19 animals and an average national herd size of 14 animals [55]. Smaller goat farms with fewer animals contributing to the sample pools thus had to be included to meet the required number of participating farms. Despite this limitation, it can, however, be assumed that the prevalence of rumen flukes in goats is indeed very low in Germany. The observed difference in prevalence between goats and sheep, as well as cattle, is also reflected in previous studies [10,38,39,42,50]. A possible explanation is the different feeding behavior of the three ruminant species. Whenever possible, goats prefer to selectively browse instead of grazing [72,73], and the grazing behaviour of cattle is even less selective than that of sheep [74]. Metacercariae of *C. daubneyi* are located closer to the ground, while those of *F. hepatica* prefer the grass tips [75]. Grazing cattle and sheep are therefore more likely to ingest rumen fluke metacercariae than browsing goats. It remains to be determined if there are any true differences in the susceptibility to rumen fluke infections between the three ruminant species. The observed differences in *F. hepatica* prevalence between sheep and goats may be explained by the significantly more frequent use of fasciolicides on the examined sheep farms, thus potentially leading to lower flock or within-flock infection and, consequently, less frequent detection of *F. hepatica* eggs in the ovine samples.

The identification of *C. daubneyi* as the predominant rumen fluke species agrees with the findings of other recent European studies [8,10,39,43,44]. As species identification was not possible for one rumen-fluke-positive farm, we can, however, not rule out the potential presence of other species, particularly *P. leydeni*, which has been identified in Irish sheep [8] and has also recently been reported in cattle from Lower Saxony [6] and Bavaria [7].

The geographical distribution of rumen and common liver flukes across the two federal states agrees with recent findings in cattle [6]. One possible reason for the inverse prevalence of rumen and common liver fluke infection in the two regions is the competition of miracidiae of *F. hepatica* and *C. daubneyi* in their intermediate host. If snails are concurrently infected with both trematodes, one species usually dominates [76]. Jones et al. [39] also detected a negative correlation between infection levels of the two fluke species in cattle herds.

Within the two German federal states, the distribution of the three trematode species followed a clear geographical pattern (Figure 3). The *G. truncatula*-dependent rumen and common liver fluke infections were primarily identified in the north of Lower Saxony and the south of Bavaria, regions known to have a coastal climate and wet pastures including marshes and flood plains (northern Lower Saxony) or very high rainfall (southern Bavaria) [77]. This geographical distribution is thus not surprising, as these regions favor habitats for *G. truncatula*.

Animal movements have been discussed as an important factor in spreading rumen flukes [39,78,79]. For example, *C. daubneyi* is likely to have been introduced to the United Kingdom due to increased animal imports following foot and mouth disease [39]. Forstmaier et al. [6] assume that the higher rumen fluke prevalence in cattle in northern Germany is associated with the more active international animal trade in the north as opposed to the south of Germany, where local breeds such as German Simmental are predominant. The distribution in sheep seems to follow this geographical trend, and this may be an indication that cattle play a role in the infection of sheep. Interestingly, co-grazing with other ruminants, predominantly cattle, was identified as a predictor for rumen fluke infections, while no statistically significant association was observed for the two liver fluke species. Liver fluke infections have long been established in small ruminants in Germany [50,80], so they have probably reached a steady state in these species. In contrast, it is likely that rumen fluke infections were initially introduced to Germany by cattle imports and have subsequently spread to sheep and that cattle still play an important role in the propagation of this parasite. In contrast to the United Kingdom [52], a steady state for rumen fluke infections has most likely not yet been reached in Germany, and we expect an increasing prevalence in cattle as well as small ruminants in the future.

No comparable studies have so far been conducted to assess the prevalence of *D. dendriticum* in small ruminants in Germany. Previous studies in goats included only low farm numbers [41], individual samples from a certain region [50] or exclusively dairy goats [48], which are commonly grazed on improved pastures to satisfy their metabolic needs and thus less likely to be exposed to habitats favoring the intermediate hosts of the lancet fluke. Similar limitations regarding sample populations apply to previous studies in German sheep, which reported *D. dendriticum* infections in 31.1% of examined sheep flocks in a certain region [81] or in 100% of examined sheep from a single flock [82].

The majority (71.9%) of lancet-fluke-positive sheep farms and goat farms were located in the northern and western parts of Bavaria, coinciding with low rainfall regions [77] and the presence of a low limestone mountain range presenting ideal habitats for the two intermediate hosts [34,83]. However, *D. dendriticum* was also identified in some moderate or high rainfall regions in southern Bavaria (24.6%) and northern Lower Saxony (3.5%).

## 5. Conclusions

Rumen fluke infections with *C. daubneyi* are established in sheep in Germany with a currently low flock prevalence. Despite anecdotal evidence of infected goats, patent rumen fluke infections were not detected in any of the examined goat herds. The use of small ruminant pastures by other ruminants was identified as a positive predictor for patent rumen fluke infections in sheep. The geographical distribution of *F. hepatica* and *D. dendriticum* infections followed a pattern reflecting the presence of suitable habitats for the intermediate hosts. Many questions regarding the epidemiology and biology of rumen flukes remain unanswered and need to be addressed in future research. It is particularly interesting to monitor any potential future changes in rumen fluke prevalence and species distribution in Germany, as well as potential effects on animal health and production.

## Figures and Tables

**Table 1:** Reported occurrence of rumen fluke infections in small ruminants in Europe specifying the number of flocks (**a**) or number of individual animals (**b**) examined, as well as the diagnostic methods used.

(a)	Country (Region)	Host Species	Method	Percentage of Positive Flocks (*n*)	Species (Identification Method)	References
	Ireland	Sheep	FEC	77.3% (304)	*C. daubneyi*, *P. leydeni* (MOL)	[8]
	United Kingdom (Wales)	Sheep	FEC	42.0% (90)	*C. daubneyi* (MOL)	[39]
	Italy (Apennines)	Sheep	FEC	16.2% (197)	*C. daubneyi* (n.s.)	[40]
	Italy (Basilicata)	Sheep	FEC	7.9% (682)	*C. daubneyi* (n.s.)	[38]
		Goats	FEC	2.7% (73)	*C. daubneyi* (n.s.)	[38]
	The Netherlands	Sheep	FEC	8.0% (489)	not identified	[10]
	Germany	Goats	FEC	2.1% (48)	not identified	[41]
**(b)**	**Country** **(Region)**	**Host** **Species**	**Method**	**Percentage of** **Positive** **Individuals (*n*)**	**Species** **(Identification Method)**	**References**
	The Netherlands	Sheep	PM	4.9% (41)	*C. daubneyi* (MOL)	[10]
	Spain (Galicia)	Sheep	FEC	0.7% (1697)	not identified	[42]
		Goats	FEC	0.0% (103)	not identified	[42]
	France (Quercy)	Goats	PM	11.5% (26)	*C. daubneyi* (HIS)	[43]
	Germany (Schleswig-Holstein)	Sheep	FEC	3.8% (474)	*C. daubneyi* (MOL)	[44]

Abbreviations: FEC, fecal egg counts/coproscopical methods; PM, postmortem examination/abattoir study; MOL, molecular species identification; HIS, histological species identification; *n*, number of examined farms/individuals; n.s., not specified.

**Table 2:** Reported occurrence of *F. hepatica* infections in small ruminants in Europe specifying the number of flocks (**a**) or number of individual animals (**b**) examined, as well as the diagnostic methods used.

(a)	Country (Region)	Host Species	Method	Percentage of Positive Flocks (*n*)	References
	Ireland	Sheep	FEC	45.9% (305)	[14]
	Ireland	Sheep	FEC	61.6% (73)	[45]
	United Kingdom (Wales)	Sheep	FEC	54.0% (90)	[39]
	The Netherlands	Sheep	FEC	49.3% (489)	[10]
	Spain (Castilla y León)	Sheep	FEC	59.3% (110)	[46]
	Italy	Sheep	FEC	7.9% (89)	[45]
	Switzerland	Sheep	FEC	4.0% (199)	[45]
	Greece (Thessaly)	Sheep	COPRO	20.0% (40)	[47]
		Sheep	SERO	85.0% (40)	[47]
		Goats	COPRO	12.0% (34)	[47]
		Goats	SERO	70.1% (34)	[47]
	France (Poitou-Charentes)	Goats	FEC, PM	5.7% (81)	[32]
	Germany	Goats	FEC	2.1% (48)	[41]
	Germany (Bavaria)	Goats	FEC	10.8% (37)	[48]
**(b)**	**Country** **(Region)**	**Host Species**	**Method**	**Percentage of Positive** **Individuals (*n*)**	**References**
	Spain (Galicia)	Sheep	FEC	6.1% (1697)	[42]
		Goats	FEC	0.0% (103)	[42]
	Greece (Thessaly)	Sheep	COPRO	11.3% (346)	[47]
		Sheep	SERO	47.3% (499)	[47]
		Goats	COPRO	3.8% (234)	[47]
		Goats	SERO	15.9% (372)	[47]
	Poland	Sheep	PM	4.7% (175,160)	[49]
	Germany (diagnostic samples, mostly northern and western Germany)	Sheep	FEC	5.1% (374)	[50]
		Goats	FEC	0.0% (98)	[50]
	Germany (Schleswig-Holstein)	Sheep	FEC	13.3% (474)	[44]

Abbreviations: FEC, fecal egg counts/coproscopical methods; COPRO, Coproantigen Test; SERO, Serology; PM, postmortem examination/abattoir study; *n*, number of examined farms/individuals.

**Table 3:** Reported occurrence of *D. dendriticum* infections in small ruminants in Europe specifying the number of flocks (**a**) or number of individual animals (**b**) examined, as well as the diagnostic methods used.

(a)	Country (Region)	Host Species	Method	Percentage of Positive Flocks (*n*)	References
	Italy (Sardinia)	Sheep	FEC	51.1% (190)	[35]
	France (Poitou-Charentes)	Goats	FEC, PM	20.0% (81)	[32]
	Spain (Region of Murcia)	Goats	FEC	5.9% (84)	[51]
		Goats	PM	20.2% (84)	[51]
	Germany	Goats	FEC	2.1% (48)	[41]
**(b)**	**Country** **(Region)**	**Host** **Species**	**Method**	**Percentage of Positive** **Individuals (*n*)**	**References**
	Spain (Galicia)	Sheep	FEC	0.8% (1697)	[42]
		Goats	FEC	0.0% (103)	[42]
	Germany (diagnostic samples, mostly northern and western Germany)	Sheep	FEC	0.3% (374)	[50]
		Goats	FEC	0.0% (98)	[50]

Abbreviations: FEC, fecal egg counts/coproscopical methods; PM, postmortem examination/abattoir study; *n*, number of examined farms/individuals.

**Table 4:** Patent rumen and liver fluke infections on Bavarian and Lower Saxonian sheep (*n* = 223) and goat farms (*n* = 143) in relation to the purpose of animal husbandry.

Purpose of Animal Husbandry	Total		Negative		Rumen Flukes ^1^		*F. hepatica* ^1^		*D. dendriticum*	
	Sheep	Goat	Sheep	Goat	Sheep	Goat	Sheep	Goat	Sheep	Goat
*Agricultural purposes*										
Meat production ^2^	160	26	117	21	5	0	4	2	35	3
Dairy production ^2^	3	54	1	48	0	0	1	4	1	2
Meat and dairy production ^2^	4	13	4	12	0	0	0	1	0	0
Wool production	1	1	1	1	0	0	0	0	0	0
Landscaping only	18	20	12	16	0	0	0	1	6	3
*Other purposes*										
Pedigree breeding ^2^	23	13	22	12	0	0	0	0	1	1
Hobby flock only	10	11	8	10	0	0	1	0	1	1
Zoo	4	4	1	4	0	0	0	0	3	0
Unspecified	0	1	0	1	0	0	0	0	0	0

^1^ Co-infections included (*n* = 1), ^2^ Landscaping included.

**Table 5:** Patent rumen and liver fluke infections on German sheep (*n* = 223) and goat farms (*n* = 143) related to recent anthelmintic treatment. Treatments carried out >6 months prior to sample collection were classified as “none”.

Anthelmintic Treatment	Total	Negative	Rumen Flukes ^1^	*F. hepatica* ^1^	*D. dendriticum*
	*n*	*n*	*n*	*n*	*n*
*Sheep*					
None	82	59	2	3	18
Fasciolicides ^2^	39	34	0	0	5
Albendazole only ^3^	12	11	0	0	1
Other	97	71	1	3	21
Unspecified	5	2	0	0	3
*Goats*					
None	63	53	0	5	5
Fasciolicides ^2^	11	8	0	2	1
Albendazole only ^3^	6	5	0	0	1
Other	63	60	0	0	3
Unspecified	6	4	0	1	1

^1^ Co-infections included; ^2^ use of different drugs including albendazole; ^3^ Effective (in increased dosage) against *D. dendriticum*.

**Table 6:** Results of simple logistic regressions for the prevalence of patent rumen fluke, *F. hepatica* and *D. dendriticum* infections on sheep and goat farms by federal state.

Predictor	OR		95% CI		*p*-Value	
	Sheep	Goat	Sheep	Goat	Sheep	Goat
*Rumen Flukes*						
Bavaria (Intercept)	0.01	n.a.	0.00–0.05	n.a.	<0.001	n.a.
Lower Saxony	5.07	n.a.	0.83–30.89	n.a.	0.076	n.a.
*F. hepatica*						
Bavaria (Intercept)	0.03	0.08	0.01–0.08	0.03–0.17	<0.001	<0.001
Lower Saxony	0.45	0.47	0.08–2.70	0.06–1.84	0.383	0.306
*D. dendriticum*						
Bavaria (Intercept)	0.46	0.12	0.33–0.66	0.06–0.25	<0.001	<0.001
Lower Saxony	0.04	0.18	0.01–0.02	0.03–1.00	**<0.001**	**0.048**

Abbreviations: OR, odds ratio; CI, confidence interval; n.a., not applicable.

**Table 7:** Results of simple logistic regressions: predictors for patent rumen fluke infections in sheep flocks.

Predictor	OR	95% CI	*p*-Value
No potential habitat for *G. truncatula* (Intercept)	0.00	0.00–0.07	<0.001
Potential habitat for *G. truncatula*	8.41	0.50–142.14	0.138
No other ruminants (Intercept)	0.01	0.00–0.04	<0.001
Co-grazing with other ruminants	16.19	2.53–103.53	**0.003**
No equids (Intercept)	0.02	0.01–0.05	<0.001
Co-grazing with equids	3.70	0.41–33.80	0.244
No history of rumen fluke infections (Intercept)	0.01	0.00–0.04	<0.001
History of rumen fluke infections	48.82	6.83–348.96	**<0.001**
No history of *F. hepatica* infections (Intercept)	0.01	0.00–0.04	<0.001
History of *F. hepatica* infections	11.82	1.85–75.71	**0.009**
No history of *G. truncatula*-dependent trematode infections (Intercept)	0.00	0.00–0.04	<0.001
History of *G. truncatula*-dependent trematode infections	49.80	2.59–958.16	**0.009**

Abbreviations: OR, odds ratio; CI, confidence interval.

**Table 8:** Results of simple logistic regressions: predictors for patent *F. hepatica*-infections on sheep and goat farms.

Predictor	OR		95% CI		*p*-Value	
	Sheep	Goat	Sheep	Goat	Sheep	Goat
No potential habitat for *G. truncatula* (Intercept)	0.01	0.03	0.00–0.08	0.00–0.09	<0.001	<0.001
Potential habitat for *G. truncatula*	2.42	2.71	0.41–14.34	0.71–22.95	0.327	0.180
No other ruminants (Intercept)	0.03	0.04	0.01–0.07	0.01–0.09	<0.001	<0.001
Co-grazing with other ruminants	0.26	2.75	0.01–4.92	0.71–14.30	0.368	0.141
No equids (Intercept)	0.03	0.05	0.01–0.06	0.02–0.11	<0.001	<0.001
Co-grazing with equids	0.51	1.70	0.02–14.43	0.26–8.97	0.690	0.503
No history of *F. hepatica* infections (Intercept)	0.02	0.01	0.01–0.05	0.00–0.09	<0.001	<0.001
History of *F. hepatica* infections	2.71	8.22	0.48–15.30	1.60–42.17	0.257	**0.011**
No history of rumen fluke infections (Intercept)	0.02	0.05	0.01–0.05	0.02–0.11	<0.001	<0.001
History of rumen fluke infections	2.84	0.81	0.33–24.55	0.01–53.73	0.339	0.922
No history of *G. truncatula*-dependent trematode infections (Intercept)	0.02	0.03	0.00–0.05	0.01–0.09	<0.001	<0.001
History of *G. truncatula*-dependent trematode infections	2.87	7.45	0.48–17.19	1.47–37.80	0.246	**0.014**

Abbreviations: OR, odds ratio; CI, confidence interval.

**Table 9:** Results of simple logistic regressions: predictors for patent *D. dendriticum* infections on sheep and goat farms.

Predictor	OR		95% CI		*p*-Value	
	Sheep	Goat	Sheep	Goat	Sheep	Goat
Dry pastures (Intercept)	0.28	0.08	0.17–0.44	0.03–0.18	<0.001	<0.001
(Temporarily) wet pastures	0.92	0.64	0.48–1.76	0.12–2.38	0.803	0.511
No other ruminants (Intercept)	0.24	0.06	0.17–0.34	0.02–0.13	<0.001	<0.001
Co-grazing with other ruminants	1.73	1.91	0.76–4.00	0.50–7.7	0.181	0.312
No equids (Intercept)	0.28	0.07	0.20–0.39	0.03–0.14	<0.001	<0.001
Co-grazing with equids	0.12	1.32	0.01–2.13	0.20–6.11	0.146	0.714

Abbreviations: OR, odds ratio; CI, confidence interval.

## Data Availability

The data presented in this study are available on request from the corresponding author. The data are not publicly available due to reasons of privacy and patient confidentiality.
